# Who is ‘on-call’ in Australia? A new classification approach for on-call employment in future population-level studies

**DOI:** 10.1371/journal.pone.0259035

**Published:** 2021-11-04

**Authors:** Madeline Sprajcer, Sarah L. Appleton, Robert J. Adams, Tiffany K. Gill, Sally A. Ferguson, Grace E. Vincent, Jessica L. Paterson, Amy C. Reynolds

**Affiliations:** 1 Central Queensland University, Appleton Institute, Adelaide, South Australia, Australia; 2 Flinders Health and Medical Research Institute (Sleep Health) / Adelaide Institute for Sleep Health, Flinders University, Bedford Park, South Australia, Australia; 3 Faculty of Health and Medical Sciences, University of Adelaide, Adelaide, South Australia, Australia; Universitat de Valencia, SPAIN

## Abstract

**Background:**

On-call research and guidance materials typically focus on ‘traditional’ on-call work (e.g., emergency services, healthcare). However, given the increasing prevalence of non-standard employment arrangements (e.g., gig work and casualisation), it is likely that a proportion of individuals who describe themselves as being on-call are not included in current on-call literature. This study therefore aimed to describe the current sociodemographic and work characteristics of Australian on-call workers.

**Methods:**

A survey of 2044 adults assessed sociodemographic and work arrangements. Of this population, 1057 individuals were workforce participants, who were asked to provide information regarding any on-call work they performed over the last three months, occupation type, weekly work hours, and the presence or absence of non-standard work conditions.

**Results:**

Of respondents who were working, 45.5% reported working at least one day on-call in the previous month. There was a high prevalence of on-call work in younger respondents (63.1% of participants aged 18–24 years), and those who worked multiple jobs and more weekly work hours. Additionally, high prevalence rates of on-call work were reported by machinery operators, drivers, community and personal service workers, sales workers, and high-level managers.

**Conclusions:**

These data suggest that on-call work is more prevalent than previously recorded and is likely to refer to a broad set of employment arrangements. Current classification systems may therefore be inadequate for population-level research. A taxonomy for the classification of on-call work is proposed, incorporating traditional on-call work, gig economy work, relief, or unscheduled work, and out of hours work.

## Introduction

On-call work is typically defined as a working arrangement where employees can be called during certain periods of time to start work [1]. These on-call periods are generally categorised as either proximal (i.e., on-call workers remaining on-site) or distal (i.e., workers being called while they are at home) [2]. While many industries may have on-call components, peer reviewed on-call research typically focuses on industries that require an immediate mobilisation response, often to safety-critical scenarios [3, 4]. Research has typically focussed on these industries due to the severity of potential consequences if there is either a delay in mobilisation or a fatigue-related error, combined with the likelihood of worker fatigue. These potential consequences also encourage active support for research within industries such as emergency services (e.g., paramedics, firefighters), healthcare, and aviation [1, 5–8]. Other research also exists addressing on-call work in urgent, but non-emergency industries, including where on-call workers are required to respond immediately to outages/faults (e.g., utilities), potentially from their own home (e.g., information technology) [9, 10]. Furthermore, laboratory studies are frequently designed to mimic these high-stress work environments [11–14]. However, limited research has been performed on the nuances of on-call work arrangements outside of these traditional settings and at the population level, including how on-call work is conceptualised by those working such arrangements. It is critical for our understanding of current working time arrangements that we can correctly identify and include any non-standard on-call work in future research–so that these workers are not overlooked by governmental and/or organisational policy.

The current proportion of workers performing on-call work across Australia, Europe and the United States is estimated to be approximately 21% - 23% [15–18] and current figures suggest that rates of on-call work are increasing [19]. In Australia, the estimated proportion of employed persons working on-call (22%) is far higher than the estimated ~10% of workers classified as such in 1998 [20]. Despite the changes, little population-level information is available regarding the kinds of employment arrangements (e.g., contract type) and working time arrangements (e.g., start/finish times, degree of advance notice) that are considered ‘on-call’.

There are a range of known adverse health [1], safety [5], stress [6, 21], and sleep outcomes [22–24] for on-call workers, in addition to nation-wide economic impacts [25]. These health and safety impacts are typically seen as a result of poor/shortened sleep in on-call workers (i.e., due to receiving calls overnight and/or working non-standard hours). Furthermore, economic impacts are seen due to workplace absences, injury, and fatalities [25]. Furthermore, issues relating to perceived job control, high work demands, and limited support are reported in on-call workers [26, 27], which may have detrimental effects on psychosocial outcomes [28]. Many guidance materials are currently available from workplaces and regulatory bodies to assist in managing the risks associated with on-call work. However, much of this advice is directed at ‘traditional’ on-call working environments, including information on the fatigue risk associated with on-call work [29], how to schedule sleep between on-call shifts [30], or the provision of appropriate places to sleep during on-call shifts [31]. However, for individuals who consider their work to be ‘on-call’, but who do not fit into the traditional definition, these guidelines may be at best ineffective, or at worst counter-productive. Furthermore, current research does not provide clarity around which aspects of on-call work are directly linked to poor health outcomes. To promote the health and safety of all on-call workers, it is critical that guidance considers and addresses the range of on-call working arrangements.

This study draws on an existing population-based Australian workforce sample to describe the sociodemographic and work characteristics of self-reported ‘on-call’ workers. We anticipate that the proportion of individuals reporting on-call work will be greater than previously reported (22% in 2018 in Australia), due to an ongoing increase in on-call working arrangements [20]. Findings from this study will provide descriptive insights useful for future development of more inclusive and tailored guidance for all on-call workers. Furthermore, this population-level information on Australian on-call workers will be used to produce a practical and currently applicable taxonomy of on-call work.

## Methods

A cross-sectional online survey was undertaken using a sample of Australian adults. Key information on survey design and presentation can be seen in the Checklist for Reporting Results of Internet E-Surveys (CHERRIES) (S1 Table). Participants were recruited by Dynata (Melbourne, Australia) from a sample of 500,000 individuals aged 18 or over (n = 2044). Sampling was designed to match key Australian Bureau of Statistics (ABS) sociodemographics (see S1 Table). The current study reflects a secondary analysis of data collected within a larger Australian study. The original project was designed to understand insomnia and sleep health in the Australian population, with key findings available elsewhere [32–34].

As all participants were members of a pre-existing web-based panel, informed consent regarding possible survey completion was obtained prior to the study commencement. Participants were then informed about the duration of the current survey, and that their responses would be confidential. Participants were then required to agree to complete the survey by clicking on a ‘Go to survey’ button. Sociodemographic factors were assessed using standard questions on age, sex, residence location (metropolitan/rural), annual household income, educational status, work status, marital status, country of birth, and language spoken at home. The survey was completed in March and April 2019 using a three-stage randomization process to minimise bias [35]. Ethical approval was obtained from the Human Research Ethics Secretariat of the University of Adelaide Office of Research Ethics, Compliance and Integrity (H-2018-214).

### On-call work

Participants were asked the question ‘Are you on call for work?’. No preceding information was given on how on-call work was defined. Response options were ‘every day’, ‘specify days per month’ (where participants provided this information), ‘don’t know’, or ‘refused’. In order to determine prevalence of on-call work, any responses of ‘every day’ or participants who specified days per month were coded as ‘yes’ to being on-call for work.

### Sociodemographic characteristics

Participants were asked to self-report gender (male, female, or other), and provide their age (in years). Age was categorised as 18–24 years, 25–34 years, 35–44 years, 45–54 years, and 55+ years. These age categories were chosen to align with Australian Bureau of Statistics (ABS) population groupings. As an indicator of current relationship/domestic status, participants were asked ‘Which of the following best describes your current marital status?’. Response items were coded as ‘never/divorced/separated/widow(er)’ or ‘married/partnered’. Household income was determined from the question, ‘Before tax is taken out, which of the following ranges best describes your household’s income, from all sources, over the past 12 months?’ Ten thousand dollar income categories were provided, with gross household income reported as ≤$30,000, $30,001-$50,000, $50,001-$100,000, and $100,001+. These income ranges represent collapsed versions of the ABS population categories.

Current residential location, and language spoken at home were also assessed. Location was categorised as Metropolitan or Rural/Regional, and Language spoken at home was categorised as English or Other due to the large proportion of English-speaking households. Participants were also asked to provide education information with the question ‘Which best describes the highest educational qualification you have obtained?’. Response items were: still at school, high school or less, left after 16 and still studying, trade, certificate or diploma, or bachelor degree or higher.

### Occupational characteristics

A number of questions about occupational status were included in the survey. To be included in analyses, participants had to report they were working in the three months preceding survey completion. Work schedule was determined from the question “Thinking about the past 3 months, which of the following best describes your work schedule?”, with the following categories used for analyses: Standard office hours, early morning shifts (start before 8am), afternoons (from 3pm), evenings (from 7pm) or nights, and rotating shifts.

Participants were asked to indicate their occupation (“What is your occupation”) (free text) and (“What type of business/industry do you primarily work?”), which was subsequently categorised according to the Australian and New Zealand Standard Classification of Occupations (ANZSCO), version 1.3 [36]. Multiple job holders were identified with the question “Do you work more than one job?” (yes/no). Work hours were determined from the question “On average, how many total hours per week do you work at a job for which you are paid?”. Work hours were categorised in line with existing published work by Kivimaki et al. [37], with categories collapsed into ranges of <35, 35–40, 41–48, and >48 hours of work per week. The industry each participant worked in was classified by the Division coding within the Australian and New Zealand Standard Industrial Classification (ANZSIC) system [36].

Burden of non-standard work conditions was determined from multiple work variables (i.e., the number of non-standard work conditions the participant reported). Participants were categorized as having no non-standard work conditions if they reported being a standard day worker only. One non-standard work condition was coded if participants reported one of either long work hours, working >1 job, or a non-standard work schedule (early mornings, afternoons, evening/nights, or rotating schedules). Two or more non-standard work conditions were coded when combinations of non-standard work conditions (non-standard schedule, multiple jobs, and/or long work hours) were reported by workers.

Physical exertion or strain at work was determined from the question “In describing the activity level of your current place of employment, how much of the time would you say your job requires a lot of physical energy or exertion?”. Responses were coded as none/almost none or some/almost all/all the time.

### Data analysis

Analyses were conducted with IBM SPSS Statistics, version 26 (IBM, Armounk, NY). Pearson χ^2^ statistics were used to determine differences in working participant characteristics and sociodemographics by whether they indicated they had worked on-call in the last month.

## Results

Of 2044 survey participants, 1057 respondents indicated they were working in the three months prior to the survey (see the CHERRIES checklist in S1 Table). Of these participants, 99.1% (*n* = 1048) provided a yes/no response to the question about on-call work in the previous month, and 91.2% (*n* = 964) provided details about their typical work schedules. Analyses were conducted on participants who had worked in the three months prior to the survey, and who provided both on-call and work schedule responses (*n* = 956, 90.4% of all workers in the sample). Prevalence of at least one day of on-call in the preceding month was 45.5% (*n* = 435) in the working sample.

### Sociodemographic characteristics of workers reporting on-call in the preceding month

Participant characteristics are reported in Table 1 by on-call (yes or no). As shown in Table 1, on-call work was significantly more likely to be reported in men, young adults, and in those who were still at school, or had left after 16 and were still studying. Domestic status, household income, location, and language spoken at home did not differ by on-call work.

**Table 1:** Prevalence of on-call work within sociodemographic characteristics.

		On-call worker		
		(n = 435)		
	n*^a^*	%	95%CI	*p*
**Demographics**				
Sex				0.015
Female	223	42.0	37.9–46.2	
Male	212	49.9	45.1–54.6	
Age (years)				<0.001
18–24	70	63.1	53.8–71.6	
25–34	120	52.6	46.2–59.0	
35–44	89	35.7	30.0–41.8	
45–54	69	35.8	29.2–42.7	
55+	87	49.7	42.4–57.1	
Current domestic status				0.282
Never/Divorced/Separated/Widow	172	47.8	42.7–52.9	
Married/Partnered	263	44.2	40.2–48.2	
Gross Household Income^/^				0.087
≤$30,000	38	59.4	47.1–70.8	
$30,001 - $50,000	56	51.4	42.1–60.6	
$50,001 - $100,000	160	44.7	39.6–49.9	
$100,001+	144	44.0	38.7–49.5	
Location				0.866
Metropolitan	331	45.7	42.1–49.3	
Rural/Regional	104	45.0	38.7–51.5	
Language spoken at home				0.388
English	394	46.0	42.7–49.3	
Other	41	41.4	32.1–51.2	
Education				0.039
Still at school/left after 16 and still studying	16	80.0	59.2–92.8	
High school or less	54	40.6	32.5–49.1	
Trade	34	46.6	35.5–58.0	
Certificate or diploma	127	43.5	37.9–49.2	
Bachelor degree or higher	200	46.5	41.8–51.2	

^***a***^ Variable columns which do not add up to totals indicate missing data.

### Occupational characteristics of workers reporting on-call work in the preceding month

**Fig 1:**
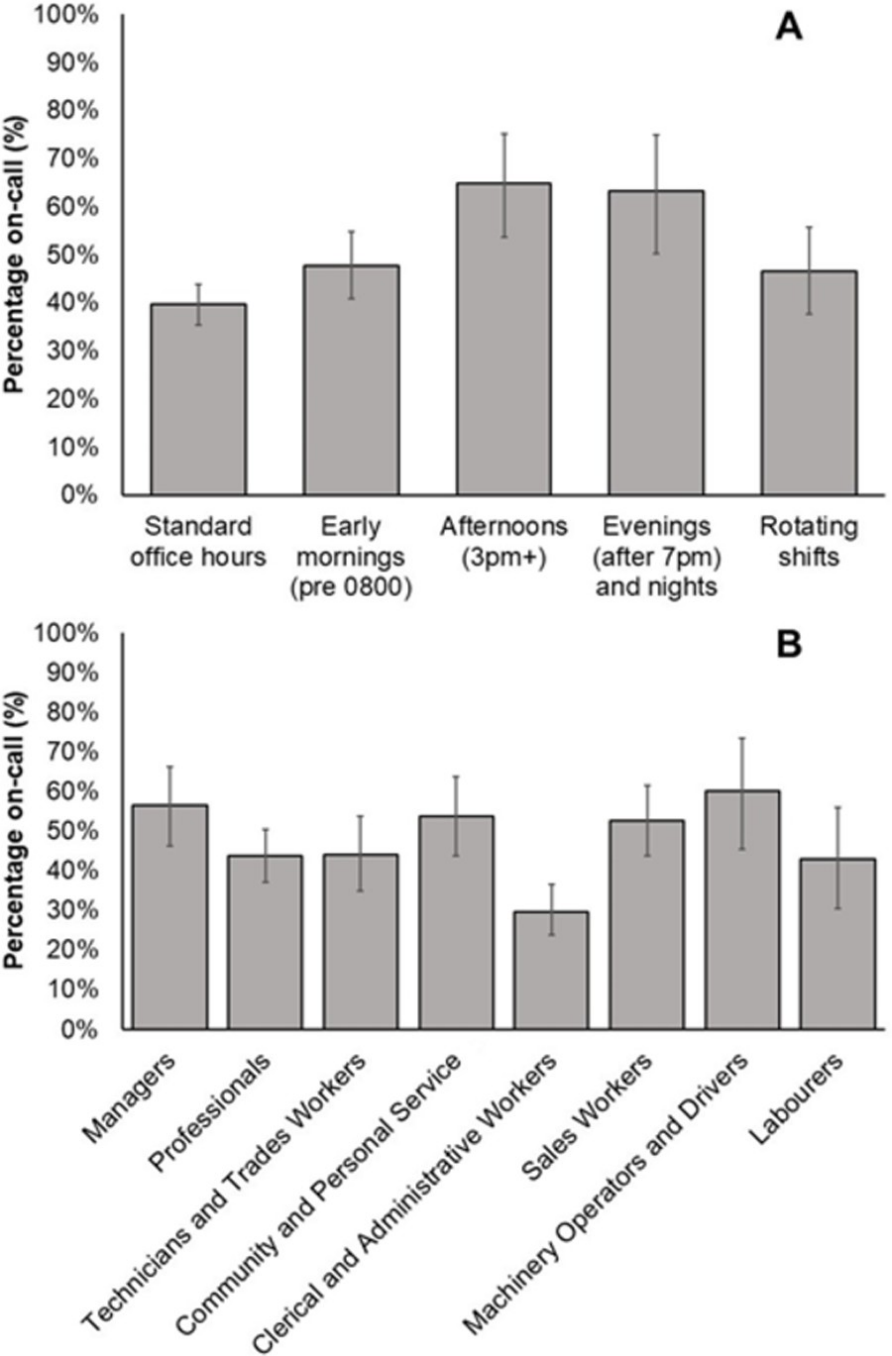
Workers reporting on-call over the previous month. Proportion (±95% Confidence Interval) of workers by work schedule (Panel A) and occupation type (Panel B) who self-reported being on-call at least once in the preceding month.

On-call conditions by work schedule and occupation type are shown in Fig 1. Reporting on-call for work in the previous month differed by both work schedule (Panel A, χ^2^_4_ = 26.0, *p*<0·001) and occupation type (Panel B, χ^2^_7_ = 33.3, *p*<0·001), with highest prevalence in those working afternoon schedules, and in machinery operators and drivers.

On-call work was associated with multiple occupational characteristics (see Table 2). In workers who reported more than one job, 62.6% (n = 72) indicated they were on-call in the last month, compared to 42.8% (n = 351) of workers who only worked one job. In workers with two or more non-standard work burdens, 62.1% (n = 113) indicated they were on-call, compared to 49.3% (n = 171) of workers with one non-standard work burden, and 35.4% (n = 151) of those with no non-standard work burden (standard day workers). Compared to those working in low physical exertion jobs, workers who reported a current job with a high requirement of physical energy or exertion were more likely to report on-call work (53.0%, n = 314 versus 33.7%, n = 117).

**Table 2:** Prevalence of self-reported on-call work by occupational characteristics.

		On-call worker		
		(n = 435)		
	n *^a^*	%	95% CI	*p*
Work more than one job				<0.001
No	351	42.8	39.4–46.2	
Yes	72	62.6	53.5–71.1	
Work hours (per week)				0.001
<35	173	47.3	42.2–52.4	
35–40	149	39.3	34.5–44.3	
41–48	31	53.4	40.7–65.9	
>48	38	64.4	51.7–75.7	
Non-standard work conditions				<0.001
Standard day worker (no non-standard)	151	35.4	30.9–40.0	
One non-standard work condition ^b^	171	49.3	44.0–54.5	
Two or more non-standard work conditions	113	62.1	54.9–68.9	
Work requires physical exertion/strain				<0.001
None/almost none	117	33.7	28.9–38.8	
Some/almost all/all the time	314	53.0	49.0–57.0	

^***a***^ Variable columns which do not add up to totals indicate missing data.

^***b***^ Long hours, >1 job, or non-standard work schedule.

The proportion of on-call workers by Division coding according to the ANZSIC system is provided in S2 Table. The industries reporting the highest prevalence of on-call work were accommodation and food services (65.2%, n = 15), manufacturing (61.4%, n = 27), agriculture forestry and fishing (56.3%, n = 9) and retail trade (56.3%, n = 72). Noting small sample sizes, low prevalence of on-call work was seen for electricity, gas, water and waste services (0.0%, n = 0), mining (25.0%, n = 1), and other services (22.2%, n = 2).

## Discussion

The findings of this study indicated that a high proportion of individuals reported engaging in on-call work (45.5% of the working sample). This figure is substantially greater than previous estimates of on-call prevalence (22%). Interestingly, just 20 of the 435 participants who reported working on-call (4.5%) were in the Public Administration and Safety classification, which includes occupations such as police, fire fighters, and other emergency services. A significant number of participants instead reported working in industries that are not typically captured in research addressing on-call work, including retail trade (16%), administrative and support services (17%), and professional, scientific, and technical services (11%). It is apparent that a number of Australians perform work they themselves consider ‘on-call’, despite not being employed in those industries that have been the focus of on-call research (e.g., emergency services, healthcare, etc.). This far exceeds our expectation based on existing literature [20] of the diversity of who considers themselves to work on-call. This may suggest that people’s understanding of what is meant by ‘on-call’ may vary from how this term is interpreted within the peer reviewed literature. Furthermore, it must be noted that one area in particular where on-call work might be expected (electricity, water, and gas) had no respondents reporting on-call work. This could indicate that there are far fewer individuals working on-call in this area than previously understood, or that our sampling methodology did not capture these individuals.

The current analyses indicated that the proportion of individuals who report on-call work differs based on occupation type. The occupation classification with the highest proportion of reported on-call work was machinery operators/drivers. Jobs performed by this group may include managing, operating, and maintaining plant and equipment, in addition to transporting passengers and freight. This occupation classification is therefore likely to include individuals who are required to be on-call within an industrial environment (e.g., responding to mechanical faults) and those who drive in an on-call capacity (e.g., Uber, Lyft, Ola, or DiDi drivers). As specific occupation was not classified, it was not possible to determine whether the high rate of on-call work in this group reflects a high proportion of on-call drivers, or a combination of drivers and plant/machinery operators. Regardless, not all workers in this category would be considered ‘traditional on-call’, or the target of most current on-call research.

A high proportion of on-call work in the preceding month was seen in the management population. This code reflects upper management only (e.g., Chief Executive Officers, Chief Financial Officers, etc.). Reported on-call work in this population may reflect on-demand responses to critical organisational issues. This may occur via email or phone outside of work hours, as opposed to unscheduled shifts (i.e., those in executive roles are unlikely to have specific ‘on-call’ hours) [38]. High job demands, such as imminent deadlines and a subsequent increase in non-standard work hours, have been described as ‘involuntary flexible work’ [39] in such roles. As may be expected, on-call work was prevalent in community and personal service workers (e.g., hospitality and carer populations), and individuals in sales–which may reflect the casualised nature of these workforces. Additionally, administrative and clerical workers (as identified by the ANZSIC system) did not report a great deal of on-call work, which is likely to reflect standard work practices and traditional office hours.

Our findings indicate that individuals reporting on-call arrangements are more likely to report working multiple jobs than those who do not work on-call. Working multiple jobs is generally associated in other studies with a higher degree of both job and financial insecurity [40]. The findings of the present study are consistent with previous research, which indicates that an increase in flexibility (based on on-call or gig work) can be associated with having multiple jobs and/or chronic underemployment [41]. Furthermore, on-call workers were more likely to be younger (63.1% of workers aged 18–24 years and 52.6% of workers aged 25–34 years reported working on-call). This also reflects prior findings that a high proportion of young people are employed in casual, unstable, or contract-based roles [42]. These roles often do not provide steady hours of work, which for many workers results in unpredictability of future working hours. Many workers may therefore consider themselves as ‘on-call’, despite these roles differing from what academic literature generally considers on-call work.

In addition to factors that are indicative of job insecurity, on-call work was associated with a variety of other non-standard work practices. Afternoon and evening work were both associated with a higher prevalence of on-call periods. This was most observable in community and personal service workers, and sales workers (see S3 Table, noting small sample sizes). Similarly, the number of hours worked per week (higher weekly work hours associated with being on-call), and number of non-standard working conditions were associated with on-call work. These findings reflect the association of on-call work with flexibility, but also with work time taking up a higher proportion of highly valued social time [43], as suggested by higher rates of afternoon/evening work and a greater number of hours worked per week. This may also reflect a potential blurring of work and non-work time, particularly for individuals who perform on-call administrative work from home (e.g., emails, calls) outside ‘standard’ work hours. Given the current number of individuals working from home due to the COVID-19 pandemic [44], the likelihood for work to encroach into non-work time may be even greater than is indicated by these data which were collected prior to the COVID-19 pandemic (collected in mid-2019).

**Fig 2:**
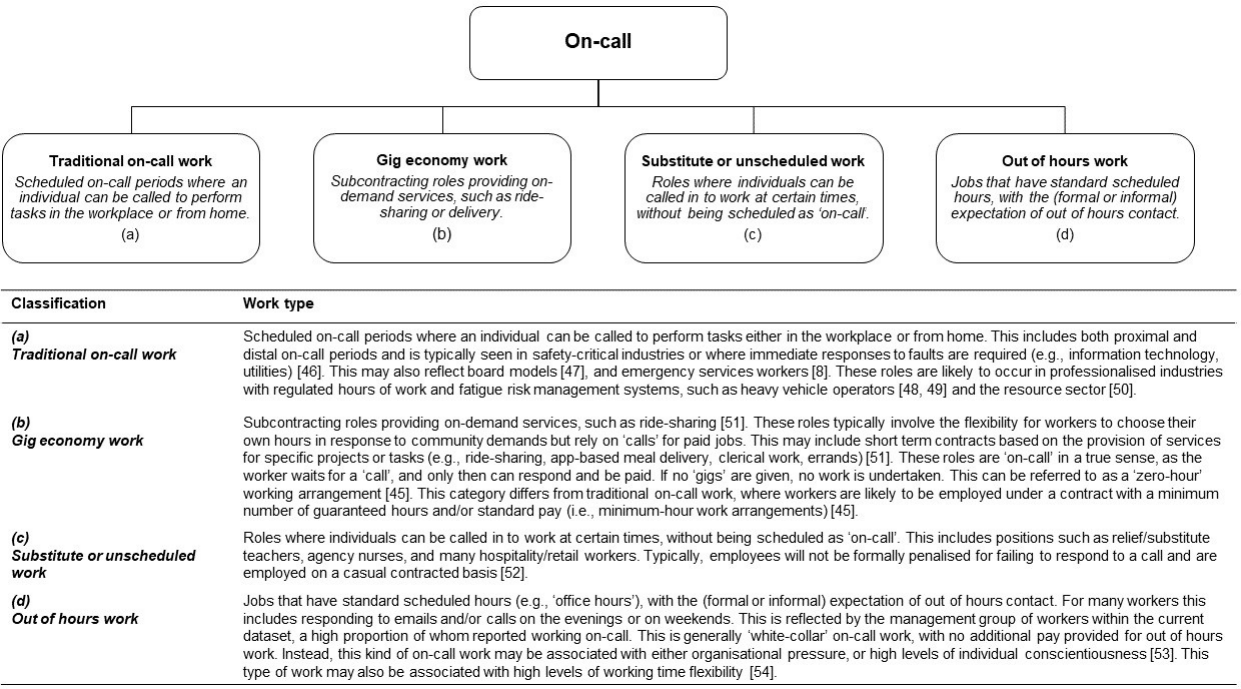
Proposed on-call taxonomy [46–54].

It is our view that future research into working time and employment arrangements should address the complexities of modern on-call work, in order to differentiate between the seemingly disparate available arrangements. The findings of this study indicate that perceived “on-call” work takes many forms and applies to a broad range of occupational groups. Current taxonomical frameworks, while giving high level classifications for broad ranges of on-call work (e.g., zero- hour or minimum-hour arrangements) [45], are as yet unable to provide a framework for in depth descriptive questions and analyses. In particular, a classification system that differentiates between the day-to-day experiences of on-call work types is required. We therefore suggest the stratification outlined in Fig 2 for use within future population level research. Furthermore, the current study indicates that within future on-call research, clear descriptions of what constitutes each working time arrangement should be provided to ensure clarity.

To address the challenges faced by each type of on-call worker, it is critical that future research differentiates between the proposed categories identified in Fig 2. For example, while a ride-share driver and a paramedic may both report working on-call, variables of interest (e.g., safety, health, performance, etc.) may be very different. A ‘one-size-fits-all’ approach to research may result in health promotion and policy strategies that do not appropriately consider individual circumstances. Unless more specific definitions of ‘on-call’ working arrangements are used, workplace hazards (e.g., fatigue) are unlikely to be identified effectively.

It is our view that these four categories provide a comprehensive classification system for different types of on-call work, for use within future population surveys. By asking participants to classify the *type* of on-call work they perform, data are likely to have additional explanatory power. We propose using this taxonomy in conjunction with hours of work information (i.e., number of hours spent on-call per week, total hours of work per week, timing of work periods, etc.), and qualitative data (to provide a deeper understanding and contextualisation of the experience of on-call). Furthermore, future research in the on-call area should include contract type (i.e., full-time, part-time, casual, subcontractor, etc.), and job insecurity (e.g., how insecure the individual feels in their employment arrangement, how consistent work hours are, and length of time spent working in an on-call role).

The lack of information on contract type and job insecurity is a limitation of the present study. Further, participants were asked to report whether they worked on-call over the preceding month. Prevalence rates presented may not be indicative of habitual on-call work rates, but rather a ‘snapshot’ of an acute timeframe. In addition, the use of ANZSCO classifications for occupation type is a limitation. While this classification system is currently the best available option for determining work type and industry, these classifications remain collapsed at the high level required to perform population level analyses—for example, machinery operators and drivers are classified as one group, which reduces our capacity to understand each group individually. As such, future research should consider a different strategy for occupational classification.

This study reveals that a high proportion of working Australians report working “on-call”. An assessment of responses indicates that on-call work is present in virtually all Australian industries and populations. This suggests that the broad term ‘on-call’ may no longer be sufficient to capture the range of working arrangements within population level data due to the differences in working arrangements usually described as ‘being on-call’. We therefore suggest the use of a taxonomy to classify on-call work arrangements, to ensure that differences in arrangements are documented, and analyses, interventions and management strategies can be tailored.

## Supporting information

S1 Table Checklist for Reporting Results of Internet E-Surveys (CHERRIES). (DOCX)

S2 Table Proportion of on-call workers by division coding according to the Australian and New Zealand Standard Industrial Classification (ANZSIC). (DOCX)

S3 Table Unadjusted prevalence (% [n]) of workers within specific work schedules who were on call, by occupation code. (DOCX)
