# Supplementary material for: Who is ‘on-call’ in Australia? A new classification approach for on-call employment in future population-level studies
Source: PLoS One. 2021 Nov 4;16(11):e0259035. doi: 10.1371/journal.pone.0259035 (PMC8568115; doi:10.1371/journal.pone.0259035)
Supplement: S1 Table — (DOCX) [file pone.0259035.s001.docx]

**S1 Table. Checklist for Reporting Results of Internet E-Surveys (CHERRIES)**

| **Item category** | **Checklist item** | **Description** |
| --- | --- | --- |
| Design |  | Adults aged 18 years and over were randomly selected from the membership of an Australian web-based panel (>500,000 individuals). This panel is held by Dynata [previously Research Now Survey Sampling International (SSI)]. |
| Ethics | Ethics approval | Ethical approval was provided by The University of Adelaide Office of Research Ethics, Compliance and Integrity’s Human Research Ethics Secretariat (H‑2018‑214). |
|  | Informed consent | Informed consent was provided by all respondents in advance as a result of their membership of the web-based panel. Furthermore, respondents agreed to complete the survey (via clicking on a ‘Go to Survey’ button) after being informed that the content would address their health. Respondents were also informed that the survey would be 20 – 30 minutes in duration, and would be fully confidential with aggregate data reported only. |
|  | Data protection | Identifying information was not collected for any respondents, resulting in a fully de-identified dataset. The dataset is kept on password protected computers and uses only proprietary survey software and local servers. |
| Development and pre-testing |  | The survey questions are based on the 2002 US National Sleep Foundation Sleep in Adults® and the 2014 Sleep in America® survey. Additional questions were added based on the Australian 2005 Sleep in Adults survey (Hillman and Lack, 2013). Questions addressing the relationship between sleep and work were also included. Draft surveys were tested by five individuals, who provided feedback on comprehension, interpretation, memory retrieval, summarising of information and availability of appropriate responses. Relevant updates were made following this feedback. The updated survey was piloted by a sample of 300 respondents. |
| Recruitment process | Open vs closed survey | This survey was open. A cohort of adults were recruited by Survey Sampling International (Dynata). These individuals were representative of Australian populations based on information about age, gender, state of residence and socio-economic status. Recruitment to the Dynata panel occurs via banner advertisements, invitations, social media, and other online strategies. Communities of interest and/or rare populations are recruited via active strategies. All respondents go through quality screening prior to panel acceptance. |
|  | Contact mode | Three stages of randomisation were undertaken to minimise bias. Random selection was used to send respondents a blinded survey invitation. Respondents then answered a set of random profiling questions. Upon completion of these questions, respondents were then matched with this survey if they were likely to be able to complete it. |
|  | Advertising the survey | No advertising was undertaken. Online panel members were randomly selected to participate. |
| Survey administration | Web/email | Respondents were channelled via the Dynata participant interface to a web-based survey hosted by UltraFeedback (an online survey site). Multiple choice, numeric, and open response options were used. |
|  | Context | Dynata is a research services company who have a pre-existing pool of potential respondents. |
|  | Mandatory/voluntary | Participation was voluntary. Respondents were screened out only based on age. |
|  | Incentives | Participation was incentivised via standard strategies used by Dynata. Points are provided to panel members for the completion of each survey. This survey provided respondents with the point equivalent of approximately $1.50AUD. |
|  | Time/date | March and April 2019 |
|  | Item randomisation | Items were not randomised |
|  | Adaptive questioning | Branched questioning was used based on previous responses (adaptive questioning). Some items also included information populated based on previous responses. |
|  | Number of items | The survey consisted of 210 items, though due to adaptive questioning not all respondents answered all questions. The maximum number of items displayed on any page was three. |
|  | Number of screens | Approximately 70 pages were shown to each respondent (depending on adaptive questioning). |
|  | Completeness check | All items were forced response. Participants had to complete all items before progressing. Drop off was low (6%). Don’t know/not sure options were included. |
|  | Review step | Respondents could review their responses using back and forward buttons. |
|  | Unique site visitor | Digital fingerprinting and traps for geo-IP violations ensured that all respondents only completed the survey once. |
|  | View rate | Not applicable; respondents were panel members who were invited. |
|  | Completion rate | 1057 adults identified themselves as workforce participants out of 2044 completions. |
|  | Cookies used | No. |
|  | IP check | Digital fingerprinting and traps for geo-IP violations ensured that all respondents only completed the survey once. |
|  | Log file analysis | Not used |
|  | Registration | A unique login was provided for each respondent. |
|  | Handling of incomplete questionnaires | Incomplete questionnaires were not included in the final dataset. |
|  | Questionnaires with atypical timestamp | No respondents completed the survey too quickly to be removed from the dataset. The fastest completion was ~20 minutes, with no ‘straight-liners’ identified. |
|  | Statistical correction | All demographic sub-groups were captured sufficiently. Quotas for certain groups were applied partway through data collection to ensure the sampling frame appropriately represented members of the Australian public. |
