# Supplementary material for: Who is ‘on-call’ in Australia? A new classification approach for on-call employment in future population-level studies
Source: PLoS One. 2021 Nov 4;16(11):e0259035. doi: 10.1371/journal.pone.0259035 (PMC8568115; doi:10.1371/journal.pone.0259035)
Supplement: S2 Table — (DOCX) [file pone.0259035.s002.docx]

**S2 Table. Proportion of on-call workers by Division coding according to the Australian and New Zealand Standard Industrial Classification (ANZSIC)**

|  | Total workers in the sample | On-call worker | |
| --- | --- | --- | --- |
|  |  | *n* | % |
| Division A Agriculture, Forestry and Fishing | 16 | 9 | 56.3 |
| Division B Mining | 4 | 1 | 25.0 |
| Division C Manufacturing | 44 | 27 | 61.4 |
| Division D Electricity, Gas, Water and Waste Services | 6 | 0 | 0.0 |
| Division E Construction | 30 | 15 | 50.0 |
| Division F Wholesale Trade | 16 | 8 | 50.0 |
| Division G Retail Trade | 128 | 72 | 56.3 |
| Division H Accommodation and Food Services | 23 | 15 | 65.2 |
| Division I Transport, Postal and Warehousing | 42 | 23 | 54.8 |
| Division J Information Media and Telecommunications | 6 | 3 | 50.0 |
| Division K Financial and Insurance Services | 42 | 21 | 50.0 |
| Division L Rental, Hiring and Real Estate Services | 5 | 2 | 40.0 |
| Division M Professional, Scientific and Technical Services | 124 | 51 | 41.1 |
| Division N Administrative and Support Services | 214 | 75 | 35.0 |
| Division O Public Administration and Safety | 49 | 20 | 40.8 |
| Division P Education and Training | 59 | 27 | 45.8 |
| Division Q Health Care and Social Assistance | 97 | 42 | 43.3 |
| Division R Arts and Recreation Services | 21 | 11 | 52.4 |
| Division S Other Services | 9 | 2 | 22.2 |
