# Supplementary material for: Who is ‘on-call’ in Australia? A new classification approach for on-call employment in future population-level studies
Source: PLoS One. 2021 Nov 4;16(11):e0259035. doi: 10.1371/journal.pone.0259035 (PMC8568115; doi:10.1371/journal.pone.0259035)
Supplement: S3 Table — (DOCX) [file pone.0259035.s003.docx]

**S3 Table. Unadjusted prevalence (% [n]) of workers within specific work schedules who were on call, by occupation code.**

|  |  | **Work schedule type** | | | | |
| --- | --- | --- | --- | --- | --- | --- |
|  | Total number on call (n) | Standard office | Early mornings (pre 0800) | Afternoons (3pm+) | Evenings (after 7pm) and nights | Rotating shifts |
| Managers | 53 | 62.3 [33] | 20.8 [11] | 3.8 [2] | 7.5 [4] | 5.7 [3] |
| Professionals | 92 | 57.6 [53] | 17.4 [16] | 9.8 [9] | 9.8 [9] | 5.4 [5] |
| Technicians and Trades Workers | 45 | 46.7 [21] | 33.3 [15] | 4.4 [2] | 4.4 [2] | 11.1 [5] |
| Community and Personal Service Workers | 50 | 12.0 [6] | 20.0 [10] | 20.0 [10] | 14.0 [7] | 34.0 [17] |
| Clerical and Administrative Workers | 58 | 62.1 [36] | 15.5 [9] | 8.6 [5] | 5.2 [3] | 8.6 [5] |
| Sales Workers | 62 | 50.0 [31] | 16.1 [10] | 21.0 [13] | 8.1 [5] | 4.8 [3] |
| Machinery Operators and Drivers | 27 | 29.6 [8] | 33.3 [9] | 11.1 [3] | 7.4 [2] | 18.5 [5] |
| Labourers | 24 | 20.8 [5] | 37.5 [9] | 8.3 [2] | 4.2 [1] | 29.2 [7] |
